# Quantification of sympathetic hyperinnervation and denervation after myocardial infarction by three-dimensional assessment of the cardiac sympathetic network in cleared transparent murine hearts

**DOI:** 10.1371/journal.pone.0182072

**Published:** 2017-07-28

**Authors:** Teruki Yokoyama, Jong-Kook Lee, Keiko Miwa, Tobias Opthof, Satoki Tomoyama, Hiroyuki Nakanishi, Akira Yoshida, Haruyo Yasui, Tadatsune Iida, Shigeru Miyagawa, Shigeo Okabe, Yoshiki Sawa, Yasushi Sakata, Issei Komuro

**Affiliations:** 1 Department of Cardiovascular Medicine, Osaka University Graduate School of Medicine, Suita, Osaka, Japan; 2 Department of Advanced Cardiovascular Regenerative Medicine, Osaka University Graduate School of Medicine, Suita, Osaka, Japan; 3 Department of Clinical and Experimental Cardiology, Heart Group, Academic Medical Center, Amsterdam, The Netherlands; 4 Department of Medical Physiology, University Medical Center Utrecht, Utrecht, The Netherlands; 5 Department of Cellular Neurobiology, The University of Tokyo Graduate School of Medicine, Tokyo, Japan; 6 Department of Cardiovascular Surgery, Osaka University Graduate School of Medicine, Suita, Osaka, Japan; 7 Department of Cardiovascular Medicine, The University of Tokyo Graduate School of Medicine, Tokyo, Japan; Nagoya University, JAPAN

## Abstract

**Background:**

The sympathetic nervous system is critical in maintaining the normal physiological function of the heart. Its dysfunction in pathological states may exacerbate the substrate for arrhythmias. Obviously, knowledge of its three-dimensional (3D) structure is important, however, it has been revealed by conventional methods only to a limited extent. In this study, a new method of tissue clearance in combination with immunostaining unravels the 3D structure of the sympathetic cardiac network as well as its changes after myocardial infarction.

**Methods and results:**

Hearts isolated from adult male mice were optically cleared using the CUBIC-perfusion protocol. After making the hearts transparent, sympathetic nerves and coronary vessels were immunofluorescently labeled, and then images were acquired. The spatial distribution of sympathetic nerves was visualized not only along the epicardial surface, but also transmurally. They were distributed over the epicardial surface and penetrated into the myocardium to twist around coronary vessels, but also independent from the coronary vasculature. At 2 weeks after myocardial infarction, we were able to quantify both denervation distal from the site of infarction and nerve sprouting (hyperinnervation) at the ischemic border zone of the hearts in a 3D manner. The nerve density at the ischemic border zone was more than doubled in hearts with myocardial infarction compared to intact mice hearts (3D analyses; n = 5, p<0.05).

**Conclusions:**

There is both sympathetic hyperinnervation and denervation after myocardial infarction. Both can be visualized and quantified by a new imaging technique in transparent hearts and thereby become a useful tool in elucidating the role of the sympathetic nervous system in arrhythmias associated with myocardial infarction.

## Introduction

Autonomic innervation of the heart is abundant [1]. Its functions have been well investigated in physiological and pathological conditions. Measurements of serum norepinephrine level [2, 3] and iodine-123 metaiodobenzylguanidine imaging [3–7] indicate that altered function of the sympathetic nervous system is associated with adverse cardiac events in patients with heart disease.

Sympathetic nerve remodeling after myocardial infarction (MI) carries a poor prognosis, because it contributes to ventricular tachyarrhythmias [7, 8]. Nerve injury caused by myocardial ischemia results in denervation, followed by abnormal hyperinnervation due to nerve sprouting [9–13]. These abnormalities of the sympathetic nervous system after MI may not only provoke arrhythmias, but also sudden cardiac death [14–18]. Despite this information, the sympathetic nerve remodeling process remains poorly understood because it was thus far not possible to visualize the nervous network in the whole heart.

Regarding the three-dimensional (3D) distribution, only fragmentary information is available through conventional imaging methods: 1) immunostaining of heart sections only reveals nerves in thin slices; and 2) whole-mount immunostaining only demonstrates nerve distribution along the epicardial surface. Recently, in the field of neuroscience, several kinds of tissue clearing techniques such as CUBIC (Clear, Unobstructed Brain Imaging Cocktails and computed analysis) [19–21] and CLARITY (Clear Lipid-exchanged Acrylamide-hybridized Rigid Imaging/Immunostaining/In situ hybridization-compatible Tissue-hYdrogel) [22–24] have been developed to implement 3D imaging of the central nervous system, and these methods can be applied to whole-body clearing [21, 23] as well as brain clearing.

Here, we show that tissue clearing techniques enable visualization of the 3D network of cardiac sympathetic nerves and using this technique, we clarify the process of neural remodeling after MI.

## Materials and methods

### Reagents and solutions

Paraformaldehyde (PFA) (162–1665), urea (216–00185), sucrose (193–00025), and 2,2′,2″-nitrilotriethanol (145–05605) were purchased from Wako Pure Chemical Industries, Ltd. (Osaka, Japan). Polyethylene glycol mono-*p*-isooctylphenyl ether (Triton X-100) (35501–15) was purchased from Nacalai Tesque (Kyoto, Japan). N,N,N′,N′-tetrakis (2-hydroxypropyl) ethylenediamine (T0781) was purchased from Tokyo Chemical Industry Co., Ltd. (Tokyo, Japan).

The CUBIC-1 reagent was prepared as a mixture of 25 wt% urea, 25 wt% N,N,N′,N′-tetrakis (2-hydroxypropyl) ethylenediamine, and 15 wt% Triton X-100 in deionized water. The CUBIC-2 reagent was prepared as a mixture of 50 wt% sucrose, 25 wt% urea, 10 wt% 2,2′,2″-nitrilotriethanol, and 0.1% (v/v) Triton X-100 in deionized water[21]. Both CUBIC reagents were prepared and degassed just before use.

### Animals

C57BL/6J mice were obtained from Charles River Laboratories Japan Inc. (Yokohama, Japan) and used in the experiments. All animal procedures were performed conform the National Institutes of Health guidelines (Guide for the Care and Use of the Laboratory Animals). All experiments were approved by the Osaka University Institutional Review Board and performed under the guidelines of the Osaka University Committee (Approval number: 23-031-026). Animals were fed ad libitum with standard rodent chow and water, under conditions of ambient temperature (23 ± 1.5°C) and a 12-hour light/ dark cycle in the institutional specific pathogen-free housing. All efforts were made to minimize the suffering of the animals.

### Myocardial infarction

Male mice (8 weeks old) were anesthetized by the inhalation of isoflurane (3% for induction and 2.5% for maintenance), orally intubated, and artificially ventilated using a constant-volume rodent ventilator (MiniVent mouse ventilator, Harvard Apparatus, Inc., Kent, UK). Left lateral thoracotomy was performed, and MI was induced by performing permanent ligation of the left anterior descending coronary artery using 8–0 Prolene (Ethicon, Somerville, NJ, USA) at a site mid between the left atrial appendage and the left ventricular apex. Myocardial ischemia was confirmed by regional wall motion abnormality and visual change of color in the region distal to the site of ligation. Non-operated mice were included to provide control data. The mice were monitored every day during the experimental procedure.

### Heart tissue clearing and fluorescence labeling

Intact adult male mice (10 weeks old, n = 5) and post-MI mice (14 days; n = 5) were used in the experiments. Tissue clearing of the heart was performed in accordance with the CUBIC-perfusion protocol reported by Tainaka *et al*. [20, 21]. Mice were anesthetized by inhalation of isoflurane (3% for induction and 2.5% for maintenance) and, for transcardial perfusion, a blunted 26-gauge needle was inserted into the left ventricle through the apex. Mice were transcardially perfused with 10 mL of cold phosphate-buffered saline (PBS) containing 10 U/mL of heparin to remove the blood, 150 mL of cold 4% (w/v) PFA in PBS, 20 mL of PBS to wash out PFA, and 20 mL of 50% (v/v) CUBIC-1 reagent (1: 1 mixture of deionized water: CUBIC-1) in this sequence. Hearts were excised and continuously immersed in 30 mL of the CUBIC-1 reagent at 37°C with gentle shaking for 2 weeks. The reagent was exchanged every day during the first week and every other day during the second week. After clearing in the CUBIC-1 reagent, hearts were washed with PBS three times for 30 minutes each time at room temperature (RT) with gentle shaking, immersed in 20% (w/v) sucrose in PBS at RT, and then frozen in OCT compound (Sakura Finetek, Tokyo, Japan) at -80°C overnight. On the next day, the frozen samples were thawed, washed with PBS three times for 30 minutes each time, and subjected to immunostaining with anti-tyrosine hydroxylase (TH) antibody (rabbit polyclonal 1:200, AB152, Chemicon, Temecula, CA, USA or sheep polyclonal 1:200, ab113, Abcam, Cambridge, UK) and anti-α smooth muscle actin antibody (rabbit polyclonal 1:200, ab5694, Abcam) in 0.1% (v/v) Triton X-100, 0.5% (w/v) bovine serum albumin (BSA) and 0.01% sodium azide in PBS for 5 days at 37°C with gentle shaking. The stained samples were then washed with 10 mL of PBST (0.1% Triton X-100 in PBS) three times for 30 minutes each time at 37°C with gentle shaking and stained with fluorescence conjugated second antibodies: Alexa Fluor 488-conjugated donkey anti-sheep immunoglobulin G (IgG) (1:200, Invitrogen, Eugene, OR, USA), Alexa Fluor 488-conjugated donkey anti-rabbit IgG (1: 200, Invitrogen) and Alexa Fluor 647-conjugated donkey anti-rabbit IgG (1:200, Invitrogen) in 0.1% (v/v) Triton X-100, 0.1% (w/v) BSA and 0.01% (v/v) sodium azide in PBS for 5 days at 37°C with gentle shaking. The stained samples were then washed with 10 mL of PBST at 37°C with gentle shaking three times for 30 minutes each, immersed in 20% (w/v) sucrose in PBS, and degassed and immersed in CUBIC-2 reagent with gentle shaking at 37°C overnight. On the next day, the CUBIC-2 reagent was exchanged and the samples were further incubated for several days. Bright-field images of the heart during the clearing process were obtained using a Leica M205FA stereomicroscope (Leica Microsystems Ltd., Wetzlar, Germany).

### Image acquisition and image processing

Images of CUBIC-cleared and immunofluorescently labeled hearts were acquired using light-sheet microscopy Lightsheet Z.1 (Carl Zeiss, Jena, Germany) equipped with a 5× objective lens (EC Plan-Neofluar 5×, numerical aperture (NA) = 0.16, working distance (WD) = 18.5 mm), LSM 700/LSM 880 confocal microscope (Carl Zeiss) equipped with a 10× objective lens (EC Plan-Neofluar 10×, NA = 0.3, WD = 5.2 mm), and fluorescence microscope BZ-X700 (Keyence, Osaka, Japan) equipped with a 2× objective lens (CFI Plan Apo λ2×, NA = 0.1, WD = 8.5 mm). Heart samples were immersed in the CUBIC-1 reagent during image acquisition. Image processing and maximum intensity projections were performed using Zen software (Carl Zeiss). Three-dimensional (3D) reconstruction of Z-stacks and conversion to movies were conducted using the Zen or Imaris software (Bitplane, Zurich, Switzerland). With regard to images acquired with BZ-X700, image processing and digital stitching were performed using BZ-X analyzer (Keyence).

### Quantification of cardiac sympathetic innervation

TH immunostaining was quantified to evaluate sympathetic innervation density. Images of 3D-reconstructed cardiac sympathetic nerves, which were obtained using a LSM 700 confocal microscope equipped with a 10× objective lens, were used to perform innervation analyses. For each sample, three regions of interest (ROI: 300 μm×300 μm×300 μm) containing maximum innervation were selected, and the nerve volume and surface area were measured by contrast discrimination using a computer-assisted image analysis system (Measurement Pro application of Imaris software, Bitplane). Size criteria were used to discount any nonspecific staining of <100 voxels. These values were averaged for each sample and compared among three groups; 1) the ischemic border zone, 2) scar region in MI hearts and 3) a corresponding area in intact hearts.

### Conventional histology by whole-mount immunostaining

Histological analysis by whole-mount immunostaining was assessed in hearts collected from adult male mice. Hearts were prefixed with microwave irradiation, immersed in PBS containing 2% PFA and microwave irradiation for 20 seconds, and fixed with 2% PFA for 4 hours at 4°C. Samples were dehydrated in 50%, 75%, and 100% methanol. To block endogenous peroxidase, the fixed samples were bleached (methanol, 0.3% hydrogen peroxide) for 30 minutes at 4°C, hydrated in 100%, 75%, 50%, and 25% methanol and PBS, and finally permeabilized and blocked by incubating twice in PBSMT (PBS containing 2% skim milk, 0.2% BSA, and 0.1% Triton X-100) for 1 hour at RT. They were incubated overnight with PBSMT containing the anti-TH antibody (rabbit polyclonal 1:400, Chemicon) at 4°C and then washed in PBSMT five times for 1 hour each time at 4°C. The primary antibody was developed by incubating 1 μg/mL of the horseradish peroxidase-conjugated anti-rabbit IgG antibody over night at 4°C. After an extensive wash with PBSMT at RT five times for 30 minutes and the final 20-minute wash with PBST (PBS containing 0.1% Triton X-100) at RT, the samples were soaked in a 3,3’-diaminobenzidine substrate solution (cat. no. 1718096, Roche, Basel, Switzerland) for 5–10 minutes at RT. The enzymatic reaction was allowed until the desired color intensity was reached, and the samples were rinsed three times in PBST. Images of whole-mount immunostained hearts were acquired using a Leica M205FA stereomicroscope.

### Statistical analysis

Data are expressed as a mean ± standard error of the mean. Statistical significance for comparison among three groups was evaluated using Steel-Dwass multiple comparison tests and JMP 9.0.0 software (SAS Institute Inc., Cary, NC, USA). A value of p ≤ 0.05 was considered statistically significant. Graphs were made using GraphPad prism (GraphPad Software Inc., La Jolla, CA, USA).

## Results

### Clearance of murine hearts using the CUBIC method

**Fig 1:**
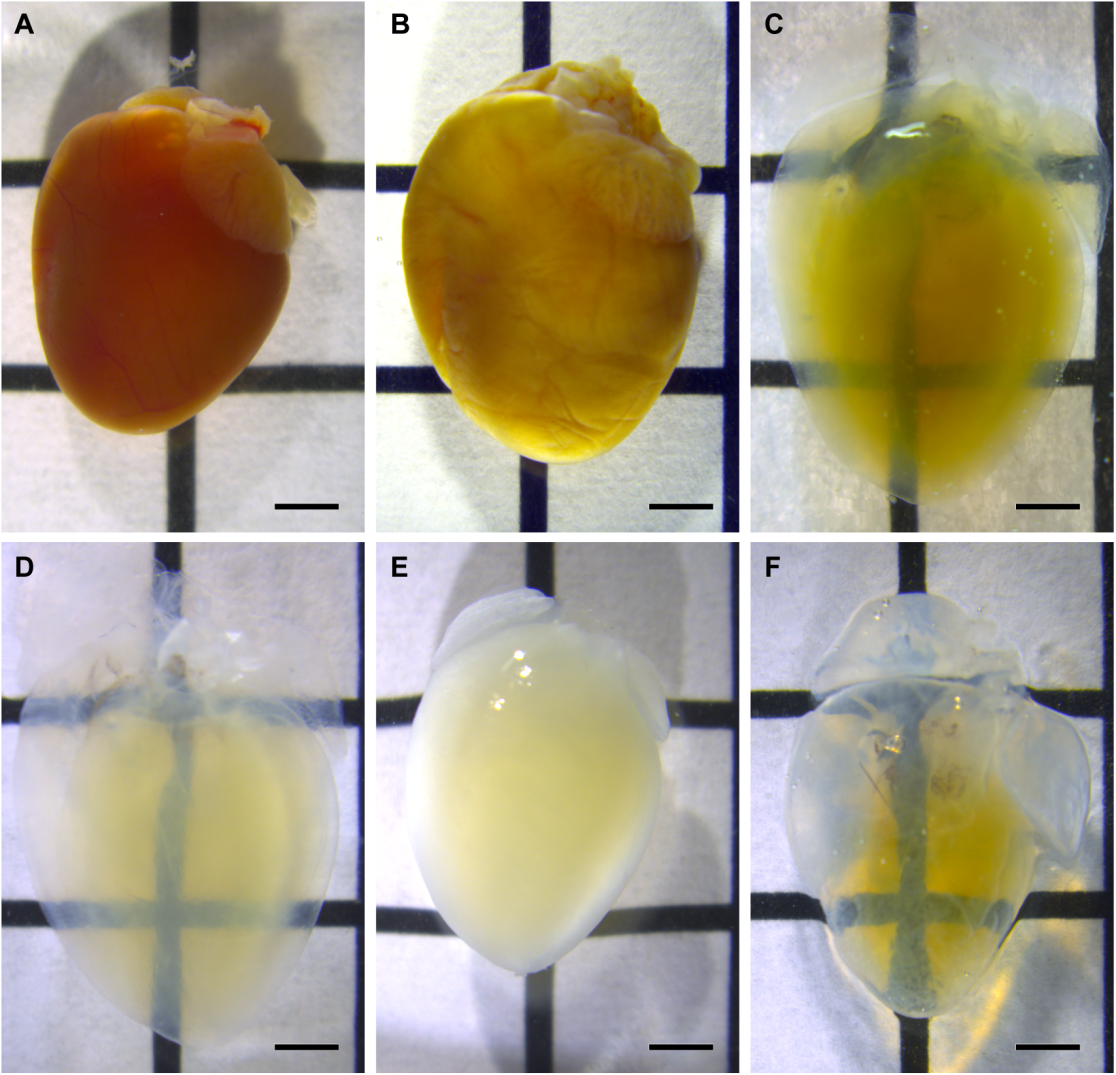
Tissue clearing process of the hearts. Bright-field images of heart samples during the clearing procedure: (**A**) After the phosphate-buffered saline wash, (**B**) after fixation with 4% paraformaldehyde, (**C**) day 1 of the CUBIC-1 process, (**D**) day 14 of the CUBIC-1 process, (**E**) after immunostaining, and (**F**) after the CUBIC-2 process. Heart samples are made transparent through the CUBIC-1 process (**A-D**). After immunostaining, samples have a decreased transparency, but the CUBIC-2 treatment restores the samples to a high transparency. Images are acquired using a stereomicroscope. The scale bars represent 2 mm.

Heart samples were transparentized and decolorized through the tissue clearing process using the CUBIC-1 reagent (Fig 1A–1D). After immunostaining with antibodies dissolved in PBS, the samples became opaque (Fig 1E). Using the CUBIC-2 reagent to match the refractive index restored the high transparency of the samples again (Fig 1F). Heart samples, which had swelled during the CUBIC-1 processing, returned to their initial size after the CUBIC-2 treatment, as was reported in the original article [19]. S1 Fig demonstrates representative images after staining with the conventional whole-mount immunostaining technique. With this conventional technique, sympathetic nerves were only observed along the epicardial surface, but not transmurally.

### Visualization of the 3D structure of sympathetic nerves and coronary vessels

**Fig 2:**
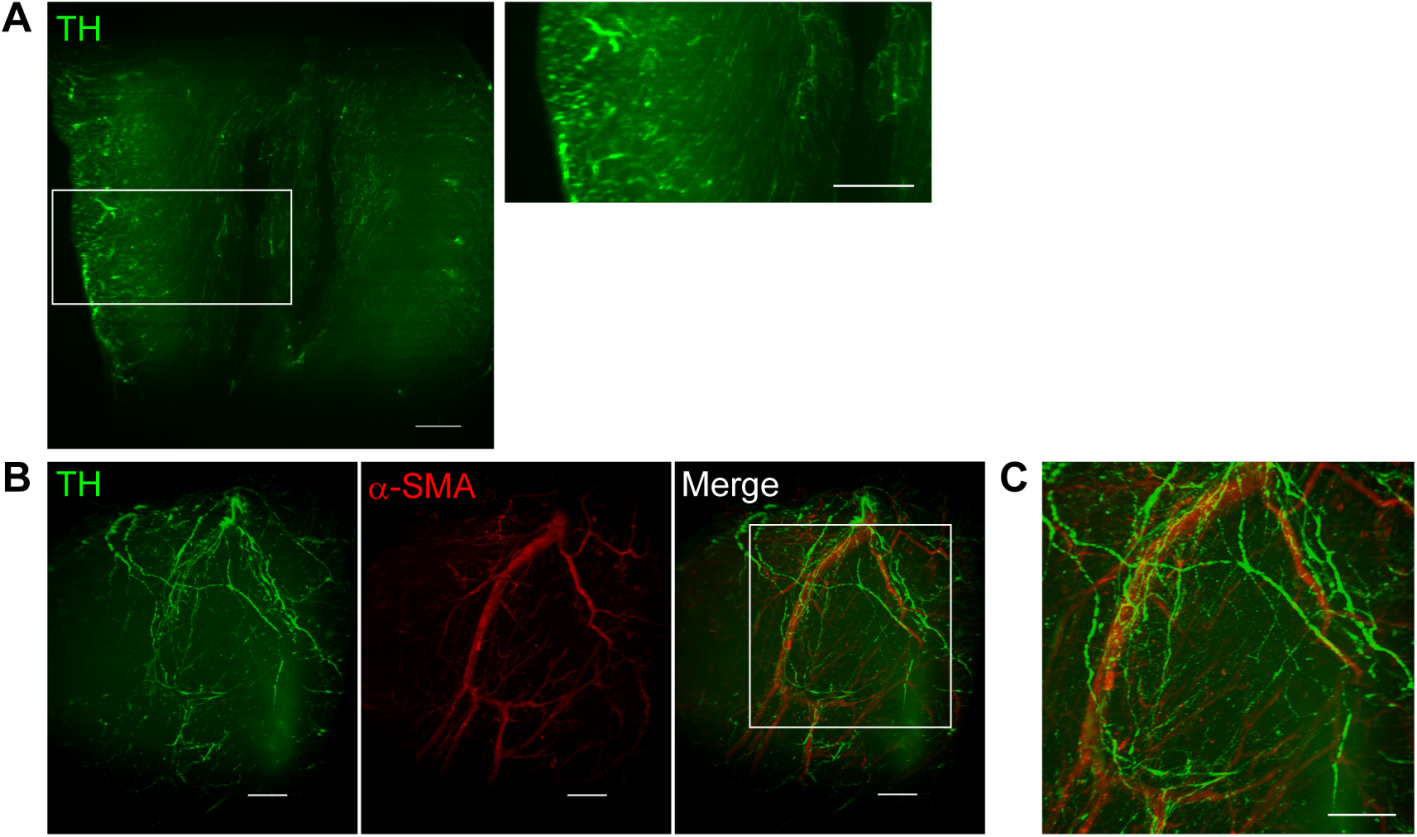
Three-dimensional (3D) distribution of sympathetic nerves and coronary vessels in the heart. (**A**) X-Y plane image of tyrosine hydroxylase (TH, green)-stained heart sample. Sufficient transparency is achieved, and the transmural distribution of sympathetic nerves is visualized. (**B**) 3D images of TH (green)-stained and α-smooth muscle actin (red)-stained heart samples. (**C**) A higher magnified view of the boxed region in **B**. Cardiac nerves distributed along the epicardial surface with nerve branches penetrating into the myocardium and wrapping around coronary vessels. Images are obtained using a light-sheet microscopy (Lightsheet Z.1, Carl Zeiss). Scale bars represent 500 μm.

**Fig 3:**
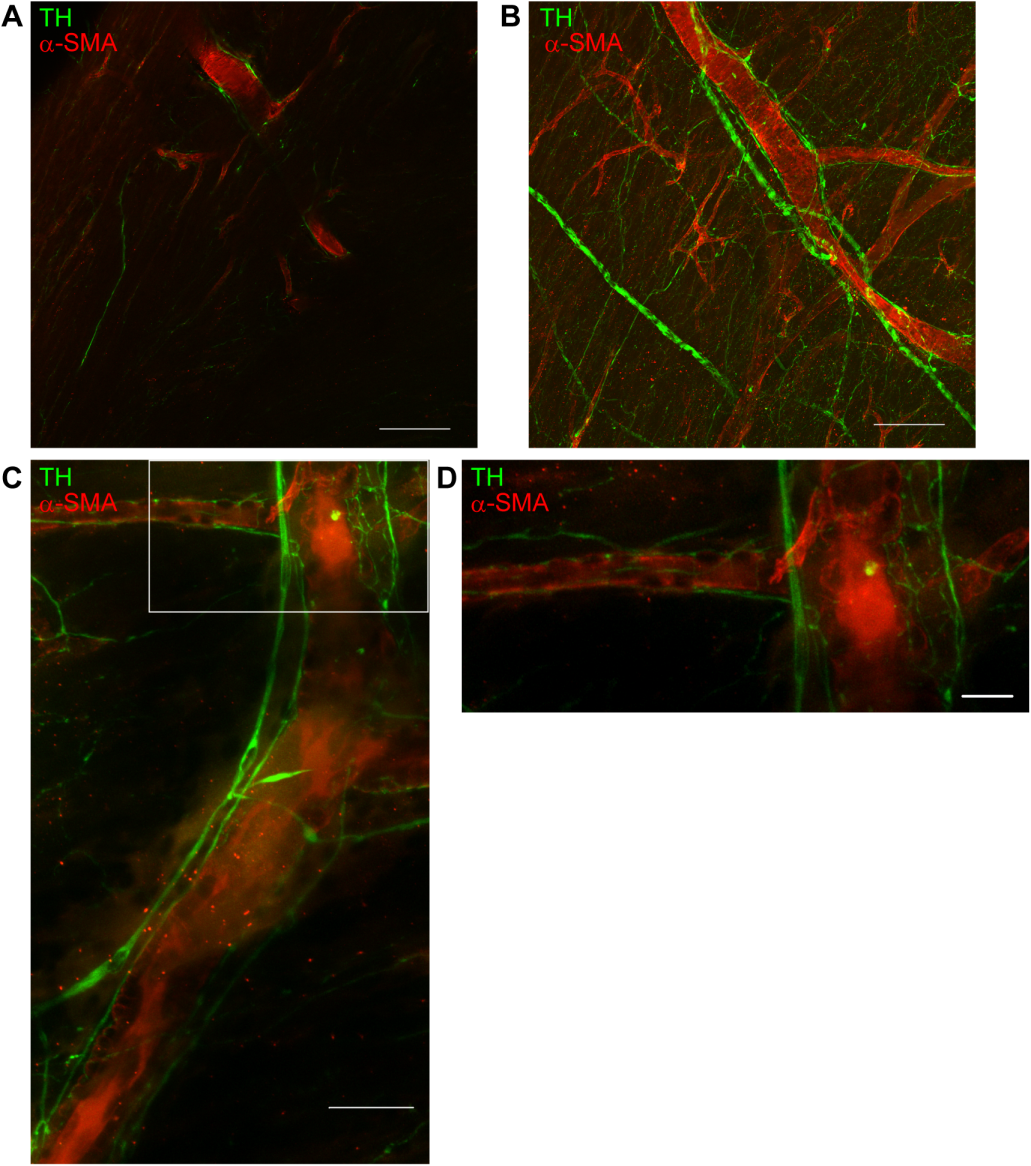
Spatial relationship between sympathetic nerves and coronary vessels. X-Y plane (**A**) and three-dimensional (**B**) images of tyrosine hydroxylase (green)-stained and α-smooth muscle actin (red)-stained heart samples. Images demonstrate that nerve fibers are distributed around coronary vessels. Images are obtained using a confocal microscope (LSM 700, Carl Zeiss). Scale bars represent 200 μm. (**C**) High resolution images of sympathetic nervous distribution around the coronary vessels. (**D**) Higher magnified view of the boxed region in **C**. Numerous hairline nerve branches are allocated around the coronary vessels. Images are acquired using a confocal microscope (LSM 880, Carl Zeiss) with Airyscan (Carl Zeiss). Scale bars represent 50 μm (**C**) and 20 μm (**D**).

Images of optically-cleared and immunostained hearts were acquired with a light-sheet microscope equipped with a 5× objective lens. Heart samples stained with the anti-TH antibody demonstrated clear images of sympathetic nerves running from the epicardium towards the endocardium (Fig 2A). In the 3D images, the spatial distribution of TH-positive sympathetic nerves and α-SMA-positive coronary vessels were visualized simultaneously (Fig 2B and 2C, S1 Video). The 3D-distribution of sympathetic nerves was investigated in more detail using the confocal microscope equipped with a 10× objective lens (Fig 3A and 3B; S2 Video). Three-D images show that the sympathetic nerves spread over the epicardial surface. From there, nerve branches penetrate into the myocardium and wrap around coronary vessels. However, there is also abundant innervation separate from the coronary vessels. Magnified images show that coronary vessels were surrounded by a network of multiple fine nerve fibers (Fig 3C). The spatial relationship between sympathetic nerves and coronary vessels is more apparent in S3 Video.

### Three-D imaging of sympathetic nervous remodeling after MI

**Fig 4:**
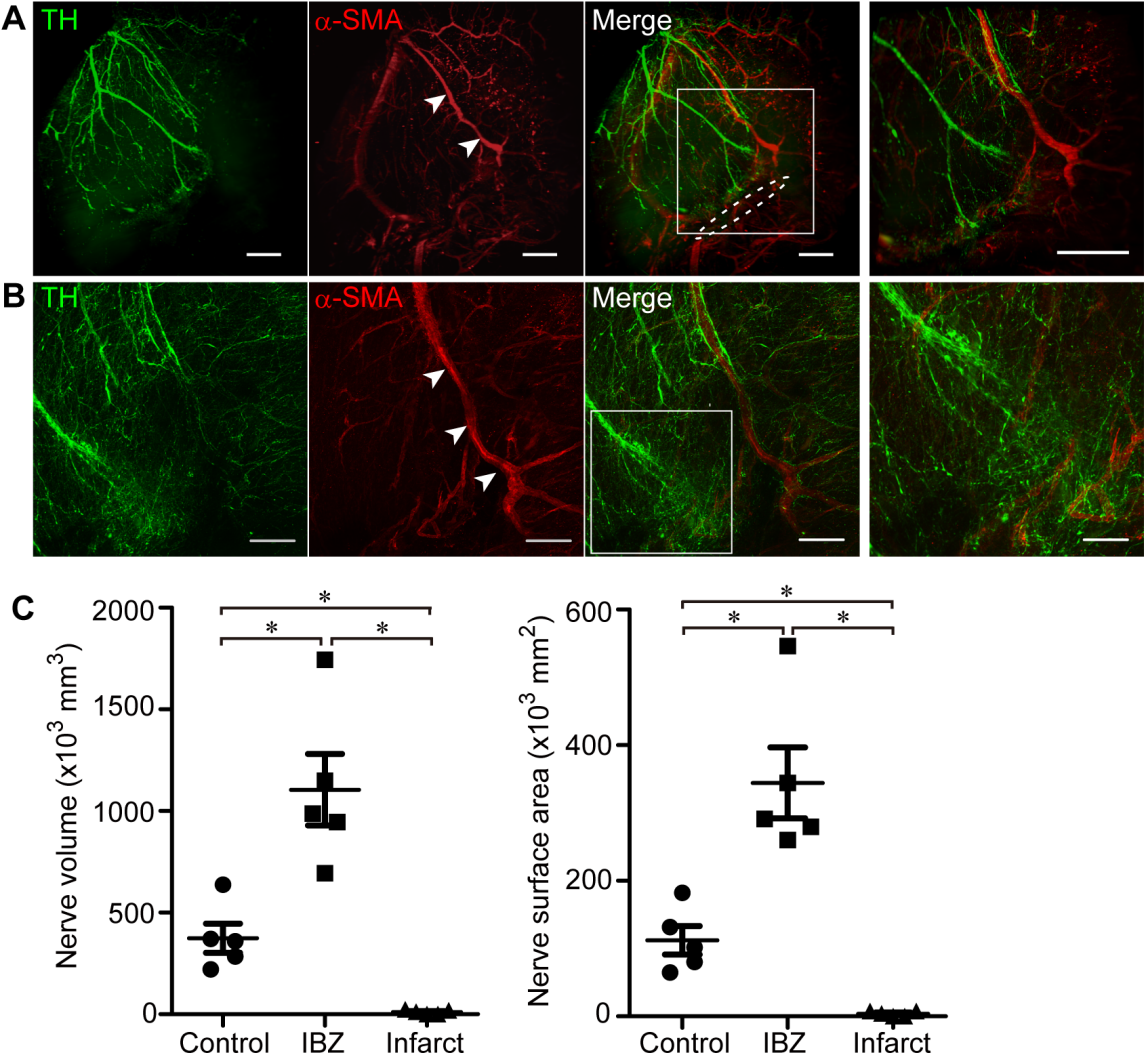
Three-dimensional (3D) imaging of sympathetic nerves and coronary vessels in the post-myocardial infarction (MI) mouse heart. (**A**) Three-D image of the heart 2 weeks after inducing MI. Heart samples are immunostained with tyrosine hydroxylase (green) and α-smooth muscle actin (red). Sympathetic nerves are abruptly extinguished at the site of the ligation. Arrowheads indicate the left anterior descending coronary artery. The dashed line indicates the site of ligation. The right panel shows a higher magnified view of the boxed region in the left panel. Images are obtained with a light-sheet microscope (Lightsheet Z.1, Carl Zeiss). Scale bars represent 500 μm. (**B**) Three-D image of the ischemic border zone in the post-MI heart. The right panel shows a higher magnified view of the boxed region in the left panel. In the ischemic border zone proximally adjacent to the site of ligation, numerous fine nerve fibers are distributed in a disordered manner. Images are obtained with a confocal microscope (LSM 700, Carl Zeiss). Scale bars represent 200 μm (left panel) and 100 μm (right panel). (**C**) Analyses of sympathetic nerves in post-MI mice. The nerve volume and surface area are significantly increased in the ischemic border zone and decreased in the infarct area of post-MI hearts compared to intact mice hearts (n = 5, *p<0.05, by Steel-Dwass test). Error bars represent standard error of the mean. IBZ, ischemic border zone.

The cardiac scar is mature and acute inflammation is resolved by 2 weeks after myocardial ischemia [25, 26]. Moreover, based on a previous report, nerve sprouting can be observed at 2 weeks after MI [13]. Therefore, we evaluated the distribution and the density of innervation at 2 weeks after MI. The same procedures of tissue clearing and immunostaining were applied in post-MI mouse hearts with comparable results concerning transparency and visibility. The spatial distribution of sympathetic nerves in the murine heart with MI was visualized (Fig 4A and 4B), and robust neural remodeling was obvious in 3D projection (S4 Video). Denervation, the abrupt extinction of sympathetic nerve fibers, was observed in the region distal from the site of coronary ligation. Thus, sympathetic nerves are scarce within the infarct area. In contrast, nerve sprouting and abnormally dense hyperinnervation was recognized at the ischemic border zone proximally adjacent to the site of ligation. Three-D analysis of cardiac nerves showed that nerve density at the ischemic border zone had increased by 2.9 times in volume and 3.1 times in surface area in hearts with MI compared to intact murine hearts (n = 5, p<0.05), whereas it had decreased almost completely in the infarct area (n = 5, p<0.05) (Fig 4C).

## Discussion

In the present study, we visualized, for the first time, the 3D structure of the cardiac sympathetic nervous network in the whole organ of adult mice (a whole organ image is available in the S2 Fig). Using the tissue clearing technique in conjunction with immunofluorescent staining, cardiac sympathetic nerve fibers and coronary vessels were simultaneously visualized.

The feasibility of heart clearing and visualization has been introduced using whole-body clearing techniques [21, 23, 27, 28]. To date, however, no paper has focused on the sympathetic nervous network of adult mice hearts. Here, we visualize by CUBIC-perfusion tissue clearing in conjunction with immunofluorescent staining the 3D structure of the sympathetic nervous network and coronary vessels not only in the normal heart, but also in hearts with MI.

In 2013, Freeman *et al*. reported *in situ* 3D imaging of sympathetic nerves in a murine heart using dopamine-β-hydroxylase (DBH)-enhanced green fluorescent protein (EGFP) transgenic mice, in which sympathetic nerves were labeled with EGFP, and image acquisition was done by two-photon excitation fluorescence microscope [29]. However, these observations were limited to the subepicardial layer (~50 μm) and only larger trunks of the nerves were observed. Much higher resolution, achieved with the present novel technique permits an overall view of the entire sympathetic network in the whole heart. This includes imaging of the peripheral fine branches and their relation with the coronary vasculature. Moreover, transmural imaging is feasible.

It has been reported that nerves course in parallel with arteries and veins in most tissues and organs, and such alignment is considered important for the mutual requirement: vessels supply nutrition and oxygen to larger nerves, whereas innervation is of crucial importance for functional control of vessels. We would like to underscore, however, that there is also abundant innervation independent from the coronary vasculature. The functional correlate is probably the sympathetic control over inotropy. Makita *et al*. reported that vascular-derived endothelins functioned as axonal guidance cues for developing sympathetic nerves in murine embryos [30]. Regarding innervation of the heart, investigations of fetal murine hearts showed the function of endothelins to guide cardiac sympathetic innervation, and sympathetic nerves extended along coronary vessels in fetal hearts [31, 32]. The present study elucidates that cardiac sympathetic nerves run along coronary vessels in adult hearts, and numerous fine nerve branches are distributed around coronary vessels, which has been assumed in the literature, but in fact never has been demonstrated due to limitations of conventional methodology.

Nerve sprouting heralds neural remodeling in MI. However, the comprehensive 3D description of this remodeling process has not been achieved. Instead, fragmentary images of nerve fibers could be observed in tissue sections. The new method of tissue clearing and software-based image reconstruction described in this study enabled us to visualize 3D sprouting of nerve fibers within a large volume of heart tissue. Furthermore, using image analysis software, we could digitize and quantify the 3D density of nerve fibers and analyze the data statistically.

Both types of abnormality in the nerve density, denervation and nerve sprouting, are related with functional change and arrhythmias. Denervation super-sensitivity elicits inhomogeneous electrophysiologic changes in the denervated area, making the heart more vulnerable to ventricular arrhythmias [14]. On the other hand, Chen and colleagues have investigated the relationship between nerve sprouting and arrhythmogenesis. In a canine model of complete atrioventricular block and MI, nerve sprouting contributes to lethal arrhythmias and sudden cardiac death [17, 33, 34]. Additionally, in patients with severe heart failure, an increased density of sympathetic nerves is associated with a history of ventricular arrhythmia [18]. Thus, evaluation of nerve density is of great significance concerning the functional aspects.

With these methods, future research in the following areas becomes feasible: (1) to investigate the contribution of innervation in myocardial regeneration [35, 36]; (2) to elucidate neural remodeling of the hearts in Takotsubo cardiomyopathy [37, 38], in atrial fibrillation and in heart failure; and, (3) to evaluate the effects of neural modulation by therapeutic strategies such as renal denervation, vagal nerve stimulation and spinal cord stimulation.

This study has several limitations. The major limitation is the systemic performance of the microscope and the attenuation of signals by light scattering. The observation range in the X-Y plane can be expanded by digital stitching using image processing software. However, the observation depth is restricted by the WD of the objective lenses. We can observe up to ~50 mm in depth using low-magnification lenses with long WD, but less by lenses with higher magnification. Attenuation of signals is inevitable even in optically-cleared samples. These limitations result in a restricted sample size. Hence, to observe whole human hearts, further technical progress is required.

In conclusion, the 3D imaging technique in a cleared heart enabled us to conduct a precise morphological analysis of the cardiac neural network. The technique is expected to be a powerful tool for investigating the role of the neural network in heart disease of several etiologies.

## Supporting information

S1 Fig Global images of cardiac sympathetic nerves without tissue clearing. Images of a whole heart immunostained with tyrosine hydroxylase using a conventional method. Images are acquired using a stereomicroscope (M205FA). (**B**) A higher magnified view of the boxed region in **A**. The scale bars represent 2 mm (**A**) and 1 mm (**B**).
(TIF)

S2 Fig Global images of cardiac sympathetic nerves in cleared heart. Frontal (**A**) and dorsal (**B**) views of the intact mouse heart immunostained with tyrosine hydroxylase (TH, green) and a-smooth muscle actin (red). In the dorsal view (**B**), ganglionated atrial plexi are demonstrated as clusters of TH-positive cells (arrowheads). Images are obtained with a fluorescence microscope (BZ-X700, Keyence) and digitally stitched. Ao, Aorta.
(TIF)

S1 Video Global images of sympathetic nerves and coronary vessels in the heart. The three-dimensional image shows that the sympathetic nerves spread over the epicardial surface of the whole organ. From these nerves, branches penetrate into the midmyocardium.
(AVI)

S2 Video Three-dimensional images of sympathetic nerves and coronary vessels in the heart. The spatial relationship of sympathetic nerves and coronary vessels is demonstrated in a three-dimensional manner. Note that many, but not all sympathetic nerves are contiguous with the coronary vessels.
(AVI)

S3 Video Magnified images of sympathetic nerves and coronary vessels. The three-dimensional architecture of sympathetic innervation to coronary vessels is visualized. Coronary vessels are surrounded by a network of multiple fine nerve fibers.
(AVI)

S4 Video Spatial distribution of sympathetic nerves in a mice heart with MI. Characteristic findings of neural remodeling in MI, i.e., denervation at distal sites from the ligation and nerve sprouting at the proximal site, can be appreciated at the three-dimensional level. MI, myocardial infarction.
(AVI)
