# Supplementary figures and images for: Quantification of sympathetic hyperinnervation and denervation after myocardial infarction by three-dimensional assessment of the cardiac sympathetic network in cleared transparent murine hearts
Source: PLoS One. 2017 Jul 28;12(7):e0182072. doi: 10.1371/journal.pone.0182072 (PMC5533449; doi:10.1371/journal.pone.0182072)

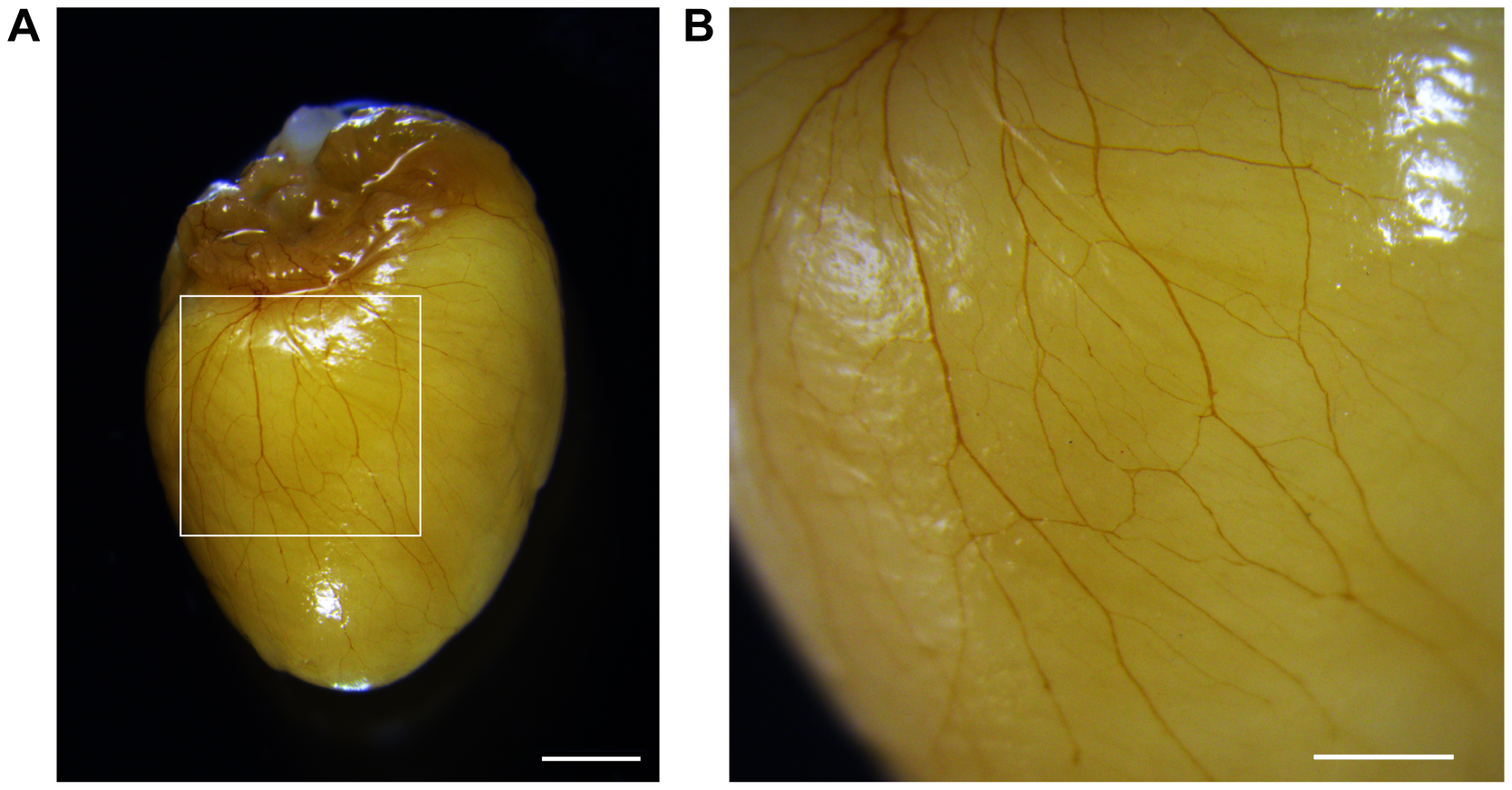

Supplement: S1 Fig — Images of a whole heart immunostained with tyrosine hydroxylase using a conventional method. Images are acquired using a stereomicroscope (M205FA). (B) A higher magnified view of the boxed region in A. The scale bars represent 2 mm (A) and 1 mm (B). (TIF) [file pone.0182072.s001.tif]

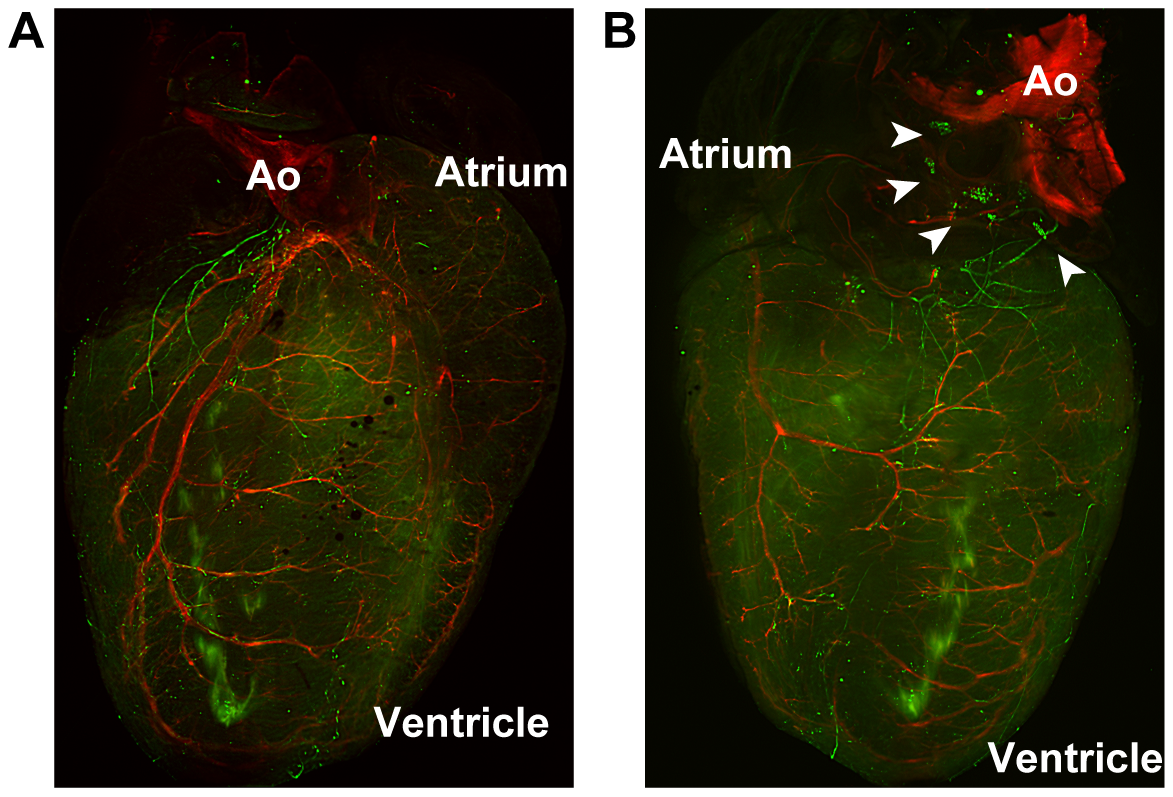

Supplement: S2 Fig — Frontal (A) and dorsal (B) views of the intact mouse heart immunostained with tyrosine hydroxylase (TH, green) and a-smooth muscle actin (red). In the dorsal view (B), ganglionated atrial plexi are demonstrated as clusters of TH-positive cells (arrowheads). Images are obtained with a fluorescence microscope (BZ-X700, Keyence) and digitally stitched. Ao, Aorta. (TIF) [file pone.0182072.s002.tif]
